# Early-phase scale-up of isoniazid preventive therapy for people living with HIV in two districts in Malawi (2017)

**DOI:** 10.1371/journal.pone.0248115

**Published:** 2021-04-01

**Authors:** Scott A. Nabity, Laurence J. Gunde, Diya Surie, Ray W. Shiraishi, Hannah L. Kirking, Alice Maida, Andrew F. Auld, Michael Odo, Andreas Jahn, Rose K. Nyirenda, John E. Oeltmann

**Affiliations:** 1 Global Tuberculosis Prevention and Control Branch, U.S. Centers for Disease Control and Prevention, Atlanta, Georgia, United States of America; 2 Center for Global Health, U.S. Centers for Disease Control and Prevention, Lilongwe, Malawi; 3 Department of HIV and AIDS, Malawi Ministry of Health, Lilongwe, Malawi; Boston University School of Public Health, UNITED STATES

## Abstract

**Background:**

Isoniazid preventive therapy (IPT) against tuberculosis (TB) is a life-saving intervention for people living with HIV (PLHIV). In September 2017, Malawi began programmatic scale-up of IPT to eligible PLHIV in five districts with high HIV and TB burden. We measured the frequency and timeliness of early-phase IPT implementation to inform quality-improvement processes.

**Methods and findings:**

We applied a two-stage cluster design with systematic, probability-proportional-to-size sampling of six U.S. Centers for Disease Control and Prevention (CDC)-affiliated antiretroviral therapy (ART) centers operating in the urban areas of Lilongwe and Blantyre, Malawi (November 2017). ART clinic patient volume determined cluster size. Within each cluster, we sequentially sampled approximately 50 PLHIV newly enrolled in ART care. We described a quality-of-care cascade for intensive TB case finding (ICF) and IPT in PLHIV. PLHIV newly enrolled in ART care were eligibility-screened for hepatitis and peripheral neuropathy, as well as for TB disease using a standardized four-symptom screening tool. Among eligible PLHIV, the overall weighted IPT initiation rate was 70% (95% CI: 46%–86%). Weighted IPT initiation among persons aged <15 years (30% [95% CI: 12%–55%]) was significantly lower than among persons aged ≥15 years (72% [95% CI: 47%–89%]; Rao-Scott chi-square P = 0.03). HIV-positive children aged <5 years had a weighted initiation rate of only 13% (95% CI: 1%–79%). For pregnant women, the weighted initiation rate was 67% (95% CI: 32%–90%), similar to non-pregnant women aged ≥15 years (72% [95% CI: 49%–87%]). Lastly, 95% (95% CI: 92%–97%) of eligible PLHIV started ART within one week of HIV diagnosis, and 92% (95% CI: 73%–98%) of patients receiving IPT began on the same day as ART.

**Conclusions:**

Early-phase IPT uptake among adults at ART centers in Malawi was high. Child uptake needed improvement. National programs could adapt this framework to evaluate their ICF-IPT care cascades.

## Introduction

Tuberculosis (TB) is the leading cause of death among people living with HIV (PLHIV), accounting for approximately 300,000 deaths among PLHIV in 2017 [1,2]. Isoniazid preventive therapy (IPT) reduces both TB incidence and mortality rates among PLHIV, independent of antiretroviral therapy (ART) [3,4], and is a criticial component of the World Health Organization’s (WHO) End TB strategy [5]. Despite long-standing recommendations for TB prevention among PLHIV, global uptake has been slow.

Malawi is among the top 20 high-burden countries for TB and HIV [2]. Malawi has nearly one million PLHIV [6], and HIV is the main driver of the TB epidemic. In 2017, of patients with TB in Malawi, 49% were PLHIV [7]. Before 2017, PLHIV in Malawi were eligible for IPT as part of a pre-ART program: patients with a new HIV diagnosis, but not yet eligible for ART per the Malawi Ministry of Health (MOH) CD4^+^ cell count threshold, began IPT and continued until ART initiation [8]. In 2015, WHO released two new guidelines that led the Malawi MOH to reconsider their TB-prevention policy for PLHIV: WHO recommended universal ART for all PLHIV regardless of CD4^+^ cell count (the *Test and Treat* strategy) and conditionally recommended at least 36 months of IPT among PLHIV in settings with high TB transmission [9,10]. The Malawi MOH revised the national guidelines to provide continuous (or lifelong), daily isoniazid with pyridoxine for all PLHIV, regardless of ART status, age, or pregnancy status, in five high-burden districts [11]. These guidelines recommend that PLHIV enroll in ART care within one week of HIV diagnosis and, if eligible, start IPT immediately. PLHIV are eligible for IPT if they screen negative for TB disease and do not have a documented contraindication (i.e., presumptive active TB defined by presence of cough, fever, night sweats, and/or weight loss [12]; active hepatitis, pre-existing liver damage, or chronic alcohol use; or severe peripheral neuropathy). Consistent with WHO guidelines, the Malawi guidelines did not require PLHIV to undergo testing for latent TB infection before initiating IPT [9]. Malawi implemented this new IPT policy in August–October 2017.

In November 2017, we assessed early IPT uptake among PLHIV newly enrolled in ART care in the early phase of programmatic implementation and scale-up, and we measured timeliness of quality-of-care indicators. We focused on the intensive case finding (ICF) and intitial IPT uptake portion of the cascade in order to direct early quality-improvement opportunities in Lilongwe district and Blantyre. We also aimed to identify gaps along the quality-of-care cascade for TB case finding and prevention among PLHIV. This evaluation derived population-based estimates for IPT uptake in the nascent stages of regional scale-up in Malawi. As global momentum builds to scale up TB prevention, similar methodology can be used to drive timely evidence-based program improvement.

## Methods

### Study setting

Collectively, the five districts where IPT was scaled up (Lilongwe, Blantyre, Thyolo, Zomba, and Chiradzulu) represented 38% of the population, 55% of patients with TB, and 40% of PLHIV on ART in Malawi according to 2016 TB and HIV program data. This study focused on Lilongwe district and Blantyre, which are among the highest density regions in Malawi. Of 245 sites treating PLHIV in these five districts, 138 (56%) were located in Lilongwe district and Blantyre, accounting for 49% of PLHIV in care.

### Study design

We measured the proportion of PLHIV newly enrolled in care (i.e., those without prior HIV treatment regardless of when HIV was first detected) who started IPT. We applied a cross-sectional, two-stage cluster medical record review at one time point in November 2017. We abstracted data from each clinic at one time point within three months of the clinic’s IPT program debut.

### Study population

For feasibility, we restricted our sampling frame to ART clinics operated by U.S. Centers for Disease Control and Prevention (CDC) implementing partners with ≥30 PLHIV currently enrolled in HIV care services. Of the 138 sites in Lilongwe and Blantyre, 28 (20%) met our criteria. These 28 sites served 64% of the PLHIV receiving ART in Lilongwe and Blantyre.

**Fig 1:**
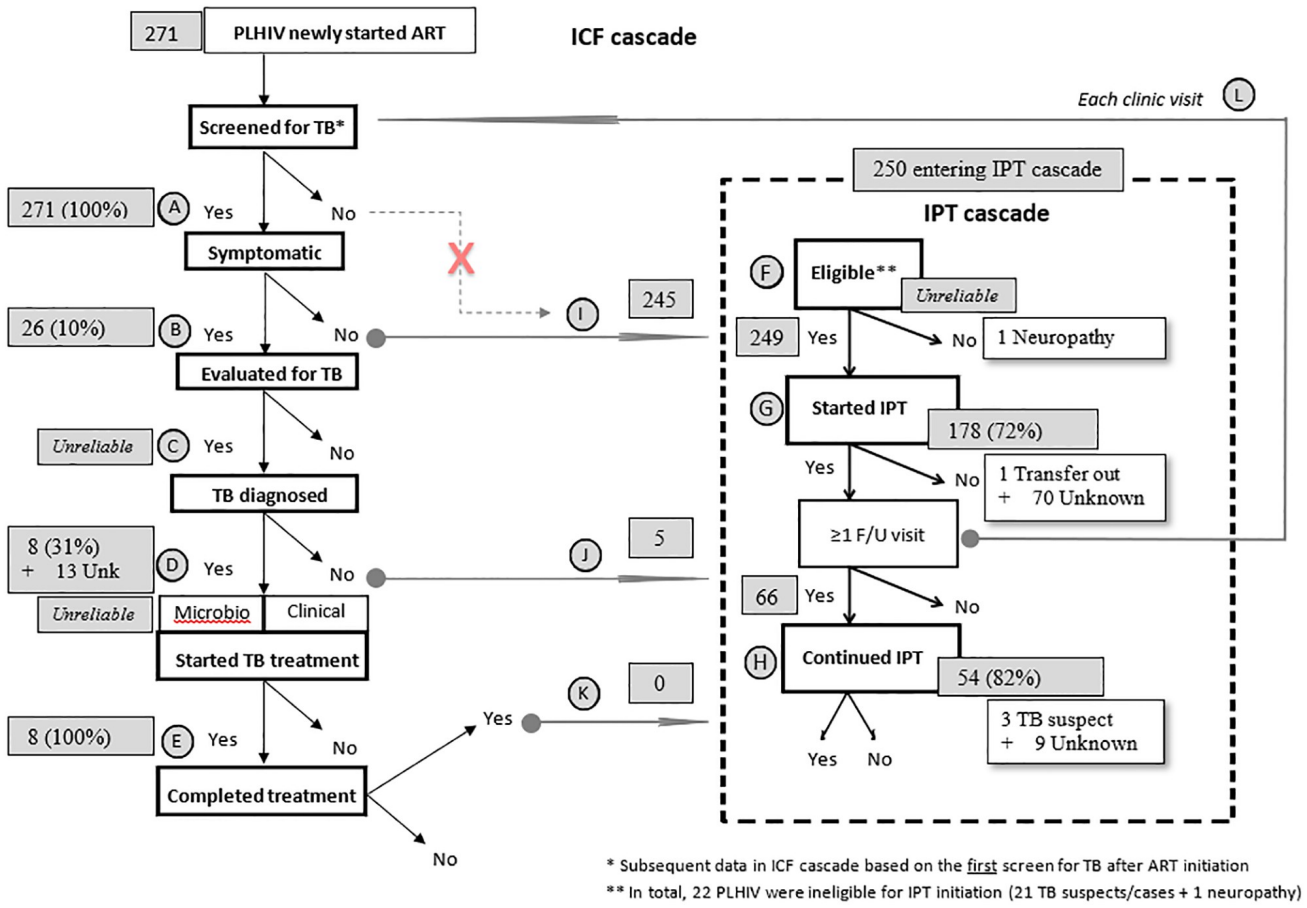
Intensified tuberculosis (TB) case-finding (ICF) and isoniazid preventive therapy (IPT) quality-of-care cascade for an unweighted sample of 271 people living with HIV (PLHIV) newly enrolled in antiretroviral therapy in Malawi (2017). Abbreviations: ART, antiretroviral therapy. Proportions are unweighted, calculated for the PLHIV sample, and were not adjusted for the two-stage, cluster-sampled design. Data in subsequent stages of ICF cascade were based on the first screen for TB after ART initiation; ** In total, 22 PLHIV were ineligible for IPT initiation (21 presumed TB [**D**] and one with pre-existing peripheral neuropathy [**F**]). **A** = Proportion of PLHIV newly enrolled in ART who were screened for TB (n = 271/271, 100% underwent four-symptom screening for cough, fever, weight loss, or night sweats). Presence of any symptom prompts a TB evaluation and withholding IPT until TB is ruled out. IPT should not be offered to PLHIV who have not undergone TB screening (**I**); **B** = Proportion of PLHIV screened for TB who were symptomatic (n = 26/271 [10%]). PLHIV with ≥1 TB symptom (i.e., positive screen) are presumed to have TB until a diagnostic evaluation rules out TB. Screened PLHIV who are asymptomatic may be eligible for IPT (**I**); **C** = Proportion of symptomatic PLHIV who were evaluated for TB (unable to calculate in this sample). PLHIV with presumptive TB must undergo a full evaluation per national guidelines; **D** = Proportion of symptomatic, evaluated PLHIV who were diagnosed with TB (n = 8/26, [31%;] 13 PLHIV had pending TB evluations at the time of this assessment). PLHIV not diagnosed with TB may be eligible for IPT (**J**); **E** = Proportion of PLHIV diagnosed with TB disease who initiated TB treamtent (n = 8/8 [100%]). Once TB treatment is completed, PLHIV may be eligible for IPT (**K**); **F** = Proportion of eligible PLHIV who screened negative for TB disease and were offered IPT (presumed 249 PLHIV were offered IPT). Programs must define eligibility status, and IPT contraindications may include a history of liver disease, viral hepatitis infection, jaundice, alcohol use, and concurrent hepatotoxic drug use. Personal interviews should determine IPT eligibility; **G** = Proportion of eligible PLHIV offered IPT who initiated IPT (n = 178/249 [71%]); **H** = Proportion of eligible, IPT-initiated PLHIV who completed (or continued) a standard course of IPT (n = 54/66 [82%] of PLHIV with ≥1 follow-up ART visit). The main IPT outcome should be documented: 1) completed/continued (i.e., ≥6 consecutive months of isoniazid or still taking isoniazid if the program recommends a longer/continuous IPT regimen) or 2) defaulted (i.e., started isoniazid and missed ≥2 consecutive months of isoniazid), including the reason for non-completion (e.g., adverse treatment event, loss-to-follow-up, or death); **I** = PLHIV with a negative TB screen may be eligible for IPT and should enter the IPT cascade (n = 245). Unscreened PLHIV should not be offered IPT until screened; **J** = PLHIV ruled out for TB who may be eligible for IPT (n = 5); **K** = PLHIV completing treatment for TB disease may be immediately eligible for IPT (n = 0); **L** = At every encounter, PLHIV should be screened for TB and re-evaluated for eligibility to continue IPT.

We employed systematic, probability-proportional-to-size (PPS) sampling based on the number of PLHIV currently in care at each clinic to obtain a reasonable estimation of IPT uptake in the target population. PPS sampling takes varying sample sizes (i.e., the number of PLHIV enrolled in care at sampled clinics) into account by weighting the sampled data, as the probability of an individual being sampled from a clinic with a large PLHIV panel is different than the probability of selection from a clinic with a smaller panel. We treated public-private status and district as implicit stratification variables. For feasibility, we limited the PPS selection to six facilities. We sampled one high-volume site with certainty because of its robust information system. We sampled the other five sites via PPS. Clients newly enrolled in ART care (i.e., persons not previously enrolled in treatment for HIV regardless of when HIV was originally detected) since the new IPT policy was implemented at each selected facility were eligible for study inclusion. We sequentially selected charts using systematic sampling, where the sampling interval (*k*) was calculated as (clinic size, N)/(target sample size, n). We aimed to sample medical records from 50 PLHIV receiving ART from each site for a total of 300 records. We used a standardized tool to abstract non-identifying, routine program data from both electronic and paper medical records. Routinely collected data elements relevant to the IPT cascade included TB symptom screening status, TB treatment history, and dates IPT was dispensed (Fig 1). Notably, we did not include reasons why PLHIV did not start or discontinued IPT, nor did we have access to detailed laboratory and clinical results for PLHIV having a positive symptom screen for TB. The monitoring and evaluation tool included only a summary assessment of whether TB disease was ruled out and whether treatment for TB was initiated. We did not collect CD4^+^ cell count at IPT initiation because this information was not routinely available and did not influence the decision to initiate IPT. Lastly, we could not reliably identify early-phase adverse treatment events because the standardized monitoring and evaluation records did not systematically include IPT outcomes.

### Ethics

The protocol and study were approved by the CDC Center for Global Health’s non-research designation review (#2017–474) and the Malawi National Health Sciences Research Committee. The review boards waived the requirement for participant consent. Only fully anonymized records were abstracted from medical records for analysis.

### Statistical analysis

Analyses were peformed using Stata/SE version 14 (StataCorp; College Station, TX). Unless otherwise noted, all analyses were weighted and controlled for the complex design of the medical record review (i.e., weighting and clustering). Sampling weights were calculated as the inverse of the product of the two-stage selection probabilties. Domain analyses were peformed on subpopulations. Statistical testing was performed using the Rao-Scott chi-square test and two-sample t-test. We considered P-values <0.05 to be significant. We calculated weighted median intervals in days between steps along the care cascade: HIV diagnosis, ART initiation, initial TB screen, and IPT initiation. We calculated IPT continuation, rather than completion, among PLHIV having ≥1 follow-up clinic visit as having a documented recurrent isoniazid prescription at their last clinic encounter. Because IPT eligibility was not explicitly noted in the medical record, we assumed PLHIV to be eligible for IPT if the initial symptom review was negative for TB disease, jaundice, and peripheral neuropathy.

### ICF-IPT cascade and quality-of-care indicators

We integrated recognized standards of care for ICF and IPT among PLHIV [13] into a quality-of-care indicator cascade. We overlaid the unweighted frequency for each indicator to recreate the progression of the sample cohort through the cascade.

## Results

### Quality of care cascade

The study sample of 271 PLHIV was representative of approximately 95,000 PLHIV in care at CDC-affiliated clinics in Lilongwe district and Blantyre (i.e., the sampling frame). The five clinics selected by PPS were, by chance, all urban, public, and equipped with electronic medical records systems. Fig 1 illustrates the logical flow of PLHIV through ART and TB clinics, which we used to measure quality-of-care indicators and to identify potential gaps. We assigned quality-of-care indicators a corresponding alphabetical designation (i.e., A‒H) as shown in Fig 1.

### Unweighted sample characteristics

Because IPT had been implemented by clinic staff less than three months prior to this cross-sectional review, three smaller clinics had not yet enrolled ≥50 PLHIV new to ART care. Our analysis cohort therefore included 271 PLHIV (90% of our planned sample). Two-thirds of the sample were women. HIV-positive women (mean age, 28.2 years [95% confidence interval (CI): 26.5–29.9 years]) were significantly younger than men (mean age, 34.7 years [95% CI: 31.6–37.9 years]; P<0.001), which mirrors the younger age distribution of HIV-positive women initiating ART nationally [14]. Of 160 HIV-positive women aged ≥15 years, 34 (21%) were pregnant, and 150 (94%) were considered eligible for IPT.

### Weighted population IPT initiation

**Fig 2:**
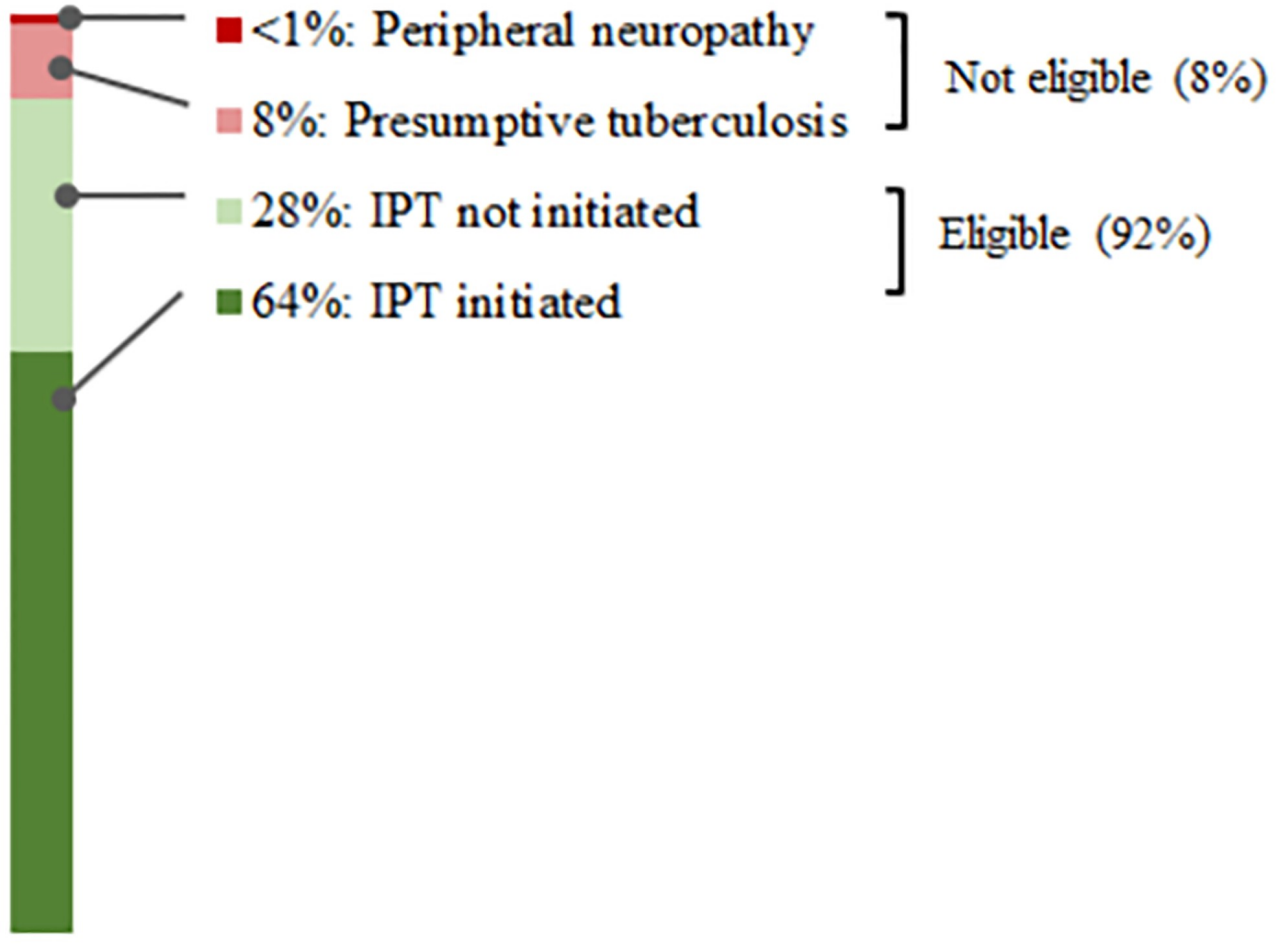
Weighted isoniazid preventive treatment (IPT) initiation rate by eligibility status for people living with HIV (PLHIV) newly enrolled in antiretroviral therapy in two urban areas of Malawi (2017).

**Fig 3:**
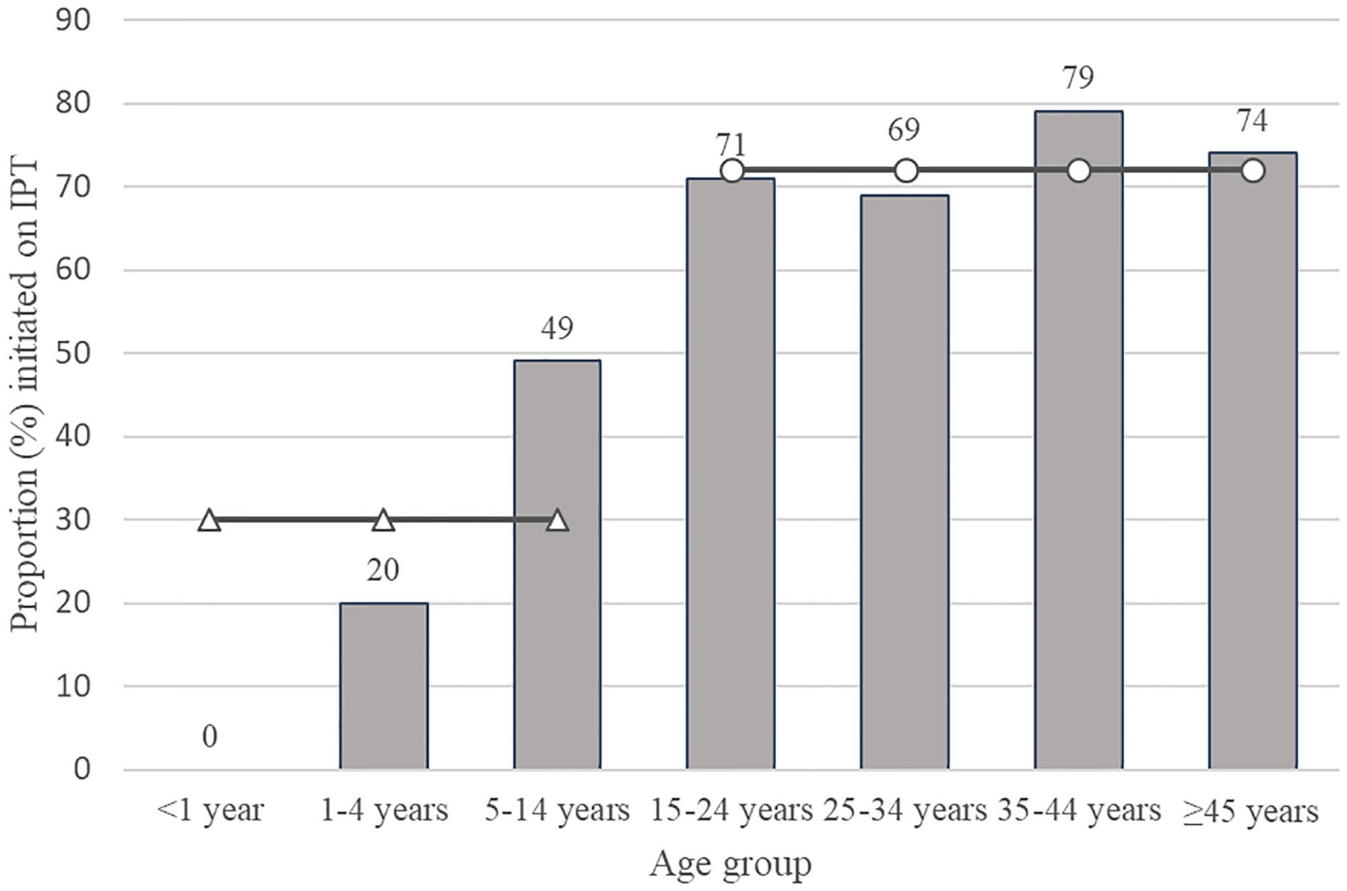
Weighted proportion of isoniazid preventive therapy (IPT) initiated among eligible people living with HIV (PLHIV) newly enrolled in antiretroviral therapy by age group in two urban areas of Malawi (2017). 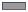
 Age group-specifc IPT initiations; 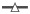 IPT initiations among PLHIV aged <15 years; 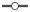 IPT initiations among PLHIV aged ≥15 years.

Of PLHIV newly enrolled in care, we considered 92% (95% CI: 84%–96%) eligible for IPT (Fig 2). Overall, 70% (95% CI: 46%–86%) of eligible PLHIV began IPT (Table 1). The proportion of eligible PLHIV aged <15 years who began IPT (30% [95% CI: 12%–55%]) was significantly lower than those aged ≥15 years (72% [95% CI: 47%–89%; P = 0.03; Fig 3). The proportion of eligible HIV-positive children aged <5 years who began IPT was 13% (95% CI: 1%–79%), and none of the four eligible children aged <2 years initiated IPT. Pregnant (67% [95% CI: 32%–90%]) and non-pregnant women aged ≥15 years (72% [95% CI: 49%–87%]) had similar IPT initiation rates (Table 1). Many PLHIV newly enrolled in care who were included in this analysis had not yet had a follow-up visit to assess adherence. We report limited treatment continuation results for 66 PLHIV: 3 (5%) at one month of follow-up, 13 (20%) at two months, and 50 (76%) at three months. Of the 66 PLHIV who initiated IPT with at least one follow-up encounter before the study team’s data abstraction, 54 (82% [95% CI: 76%–87%]) remained on treatment at follow-up.

**Table 1:** Proportion of people living with HIV (PLHIV) newly enrolled in antiretroviral therapy achieving key indicators in the isoniazid preventive therapy (IPT) quality-of-care cascade in Malawi (2017).

Quality-of-care indicator	Unweighted n/N (%)	Weighted % (95% CI)^1^	Design effect
PLHIV screened for TB	271/271 (100)	100 (97–100)^2^	NC
PLHIV symptomatic for TB	26/271 (10)	10 (5–17)	1.4
PLHIV with a TB diagnosis	8/26 (31)	27 (5–72)	2.6
Eligible^3^ PLHIV initiating IPT	178/249 (71)	70 (46–86)	6.8
Age (years)			
<5	1/8 (13)	13 (1–79)	1.1
5–14	3/6 (50)	49 (23–75)	NR
15–24	39/52 (75)	71 (50–86)	1.1
25–44	116/158 (73)	72 (42–90)	6.7
≥45	19/25 (76)	74 (48–90)	NR
Women aged ≥15 years			
Pregnant	25/34 (74)	67 (32–90)	1.9
Non-pregnant	84/116 (72)	72 (49–87)	2.8
Eligible PLHIV continuing IPT	54/66 (82)	82 (76–87)	NR

Abbreviations: CI, confidence interval; TB, tuberculosis; NC, not calculated; NR, not reported for calculated design effects <1.0.

^1^ Taylor series variance estimates for cluster sampling design.

^2^ Exact confidence limits calculated using per-protocol assignment of design effect = 2.

^3^ Eligible PLHIV excluded those with confirmed or suspected TB or with pre-existing peripheral neuropathy.

Demographics of the nearly one-third of eligible PLHIV who did not start IPT (Fig 2) were similar to the overall study population; of these patients, 67% (95% CI: 59%–74%) were women, with a mean age of 28.5 years (95% CI: 22.5–34.5 years). Most (96% [95% CI: 79%–99%]) persons ineligible for IPT had presumptive TB disease. Providers screened 99.7% of PLHIV for TB using a four-question symptom screening tool on or before the ART start date.

### Weighted population timeliness of ART and IPT initiation

Among newly enrolled PLHIV with a known HIV diagnosis date, 87% (95% CI: 70%–95%) initiated ART the same day that HIV was confirmed, and 95% (95% CI: 92%–97%) initiated ART within 1 week of confirmation. Among PLHIV without same-day ART initiation, the median delay was 3 days (interquartile range [IQR]: 1–17 days). Among eligible PLHIV, 92% (95% CI: 73%–98%) of clients started IPT on the same day as ART. For the PLHIV who did not start IPT on the same day as ART, the median delay to begin IPT was 28 days (IQR: 24–28 days), which corresponds to the first monthly follow-up visit.

## Discussion

Early-phase IPT initiations among adults with a new HIV diagnosis and newly enrolled in ART services was generally successful in urban Malawi, whereas uptake among children was low. TB symptom screening was high for all ages. Incorporating IPT into the *Test and Start* strategy resulted in timely IPT initiation for those who started IPT. Infrastructure built around ART scale-up in Malawi provided the foundation for the integration of IPT: pioneering national leadership, clear benchmarks for evaluation, uniform treatment protocols, and standardized systems coordinated by the MOH for training staff and accrediting decentralized health facilities alongside quarterly supervisions [15–17].

Ongoing programmatic enhancements targeted to improve ART service delivery likely also contributed. For example, the Malawi ART clinics used touchscreen electronic medical records that standardize clinical encounters and support decision-making [18]. Providers were required to answer TB-screening questions on the electronic medical records before moving to the next screen, and the system automatically recorded IPT prescriptions. Additionally, the Malawi MOH repeatedly engaged clinicians to address IPT hesitancy among healthcare providers and ensured a reliable medication supply chain to prevent stock outs. Finally, the decision to prioritize IPT scale-up in limited, high-burden geographic regions may have contributed to early success in Malawi.

Much of the foundational knowledge of IPT delivery, adherence, and outcomes in sub-Saharan Africa comes from controlled, experimental protocols [4,19–24]. The few programmatic reports that have focused on adherence and outcomes typically have included PLHIV who have already initiated IPT [25,26]. Because substantial attrition may occur in upstream steps of the IPT cascade (e.g. between HIV test result and TB screening) [19,27,28], including this portion of the care cascade in programmatic evaluations could help identify patients who are not receiving IPT. Monitoring PLHIV through the duration of the treatment cascade, although not part of this report, is equally important. Few studies have reported programmatic experience with the longer regimen recommended for countries with high TB prevalence [29]. One encouraging report from Eswatini showed that 36 months of IPT was feasible and had favorable outcomes among tuberculin skin test-positive PLHIV in two clinics [30].

At the time of this study, several healthcare workers reported unfamiliarity with isoniazid dosing and counseling about potential adverse treatment effects in special populations, particularly children and pregnant women. Although we saw quantitatively equivalent proportions of IPT initiation among pregnant and non-pregnant women in this urban cohort, a recent report from rural Malawi suggests IPT completion—defined by the authors as finishing a 6-month course of daily isoniazid—among pregnant women may be lower than among non-pregnant women [31]. Therefore, ongoing monitoring of IPT continuation and support for this high-risk group could help improve outcomes. IPT initiation among HIV-positive children was low; however, we did not power this medical record review for age-stratified analyses, and the 95% confidence interval had low precision. Nonetheless, this finding is important, especially because it mirrors gaps in pediatric ART coverage [32]. We hypothesize that healthcare worker inexperience or discomfort treating young children, a common programmatic experience across countries, may have contributed to the low pediatric rate [33]. Enhanced training for TB prevention among children and easy-to-use pediatric dosing tools could strengthen child IPT uptake. Additionally, focused pediatric performance measures may aid monitoring of uptake and improve program performance among children.

Our findings informed Malawi MOH’s early, focused interventions, including enhanced provider education and simpler dosing instructions. Because HIV-positive children have particular vulnerability to TB and there are added barriers to IPT implementation in this population, future operations research—with special focus on children not only within the integrated TB-HIV care cascade but also through routine contact investigation cascades in the community—is warranted [34–37].

Our study had several limitations. Our feasibility-restricted sampling frame limited generalizability of our findings to CDC-affiliated ART clinics in the two largest urban areas of Malawi. Nonetheless, these areas contain nearly one-third of in-care PLHIV in the five high-burden districts. The use of electronic health records to navigate PLHIV clinical encounters may have aided IPT initiation success. More than two-thirds of all PLHIV were served by clinics using electronic records at the time of the study. These findings may therefore not be generalizable to clinics not using electronic records at the time in Malawi. We could not collect the reasons why 28% of our eligible PLHIV population did not start IPT, but the circumstances surrounding non-initiation warrant additional scrutiny. We also focused on PLHIV newly entering HIV care and did not assess uptake among PLHIV already in care. Our evaluation did not include the laboratory components of the IPT cascade, and we could not assess long-term adherence due to the recency of IPT implementation. Nonetheless, we observed 82% continuation at short-term follow-up (i.e., within 3 months of IPT start). We could not assess whether PLHIV without a follow-up prescription for isoniazid upon our evaluation resumed IPT at subsequent visits. Routine monitoring of program data has been necessary to understand long-term IPT adherence for newly enrolled PLHIV and those already in care. Lastly, we did not power this medical record review for subgroup comparisons; therefore, some subpopulation estimates had low precision and large design effect.

The rates of IPT uptake in the early phase of scale-up were high among adult PLHIV newly enrolled in ART care in urban Malawi. Early scale-up among children needed improvement and the considerable short-term attrition warrants better understanding and long-term monitoring. The integrated Malawi IPT program may serve as a model for other countries just as the ART scale-up experience [15] and early adoption of the option B+ (i.e., preventing vertical transmission) [38] and *Test and Treat* strategies in Malawi [39] aided the global public health response to HIV. Because ART and IPT delivery integration (e.g., differentiated service delivery models [25]) was associated with higher IPT initiation and retention [20,26,40], we recommend that future evaluations include an integrated, comprehensive quality-of-care cascade. Including adverse treatment event surveillance and monitoring systems in TB-preventive treatment scale-up could help improve outcomes. Ideally, IPT monitoring and evaluation could incorporate IPT eligibility status and reasons for IPT non-initiation or premature discontinuation. TB notifications declined after ART scale-up in Malawi [41], and similar ecological analyses could follow IPT scale-up. Additional operations research that compares the features of highly successful programs with those of less-effective programs could help guide future TB-preventive treatment implementation strategies.
